# Neuropsychological task outcomes among survivors of childhood acute lymphoblastic leukemia in Malaysia

**DOI:** 10.1038/s41598-024-58128-1

**Published:** 2024-04-04

**Authors:** Hamidah Alias, Norashikin Mohd Ranai, Sie Chong Doris Lau, Leo M. J. de Sonneville

**Affiliations:** 1 Department of Pediatrics, Faculty of Medicine, The National University of Malaysia, 56000 Cheras, Kuala Lumpur, Malaysia; 2 Department of Pediatrics, Faculty of Medicine, Universiti Teknologi Mara (UiTM), 47000 Shah Alam, Selangor Malaysia; 3 Clinical Neurodevelopmental Sciences, Faculty of Social Sciences, Leiden University, Wassenaarseweg 52, 2333 AK Leiden, The Netherlands

**Keywords:** Cancer, Neuroscience, Psychology, Neurology, Oncology

## Abstract

This study intended to explore the neuropsychological ramifications in childhood acute lymphoblastic leukemia (ALL) survivors in Malaysia and to examine treatment-related sequelae. A case-control study was conducted over a 2-year period. Seventy-one survivors of childhood ALL who had completed treatment for a minimum of 1 year and were in remission, and 71 healthy volunteers were enlisted. To assess alertness (processing speed) and essential executive functioning skills such as working memory capacity, inhibition, cognitive flexibility, and sustained attention, seven measures from the Amsterdam Neuropsychological Tasks (ANT) program were chosen. Main outcome measures were speed, stability and accuracy of responses. Mean age at diagnosis was 4.50 years (SD ± 2.40) while mean age at study entry was 12.18 years (SD ± 3.14). Survivors of childhood ALL underperformed on 6 out of 7 ANT tasks, indicating poorer sustained attention, working memory capacity, executive visuomotor control, and cognitive flexibility. Duration of treatment, age at diagnosis, gender, and cumulative doses of chemotherapy were not found to correlate with any of the neuropsychological outcome measures. Childhood ALL survivors in our center demonstrated significantly poorer neuropsychological status compared to healthy controls.

## Introduction

Acute lymphoblastic leukemia (ALL) accounts for almost one third of the diagnosis in childhood cancers, making it the most common childhood malignancy^1^. Advances in treatment of ALL in the past 4 decades have significantly improved the 5-year overall survival (OS) to more than 90% in developed countries^2,3^. Modern treatment protocols, improvement in risk stratification strategies, risk-directed multiagent chemotherapy regimens and enhanced supportive care contributed to this success. In low-middle income countries (LMIC), the 5-year event-free (EFS) and OS were slightly lower at 74% and 82% respectively as reported in the Intercontinental-BFM2002 research^4^. In Malaysia, the cure rate of children with standard risk ALL is almost comparable to developed countries while those stratified to high risk (HR) group had a lower OS^5^.

Central nervous system (CNS)-directed therapy conventionally involved cranial irradiation (CI) which has now been replaced by intrathecal and systemic chemotherapy, due to the former’s many late effects^2,3,6,7^. Major chemotherapeutic agents used as CNS prophylaxis or in the treatment of CNS involvement in ALL, are methotrexate, cytarabine, and corticosteroids^2^. These drugs do, however, cause central neurotoxic adverse effects that lead to cognitive and neuropsychological dysfunction^8–13^. Previous studies of neuropsychological outcomes among survivors of childhood ALL reported deficits in processing speed, attention, working memory, executive functions and visuomotor control^14–21^. Poorer outcomes were linked to more intensive therapy, younger age at diagnosis and female gender^15,16^. Recent studies reported specific polymorphisms in neurogenesis genes which increase patients’ risk of developing chemotherapy-related neurotoxicity^22–25^.The frequencies of these polymorphisms differ between the Caucasian and Asian populations, thus the neurotoxicity risks vary even among those using the same treatment protocols. It is important to investigate the neuropsychological consequences of childhood ALL and its treatment, as they could unfavorably affect quality of life (QOL) and scholastic career^26–29^. Survivors of childhood ALL have reported lower QOL compared to healthy population with poorer school performances and emotional functioning^26^. Consequently, those with attention or memory problems, or task efficiency limitations face the risk of unemployment and were unable to live independently^30,31^. As reports on neuropsychological dysfunction in LMICs are scarce, the findings from this study may provide insight to clinicians and policymakers to plan an optimal long-term multidisciplinary care for the survivors during follow up. In this study, we also investigated whether disease-related or treatment-related factors were linked to reduced neuropsychological functioning.

## Results

**Figure 1:**
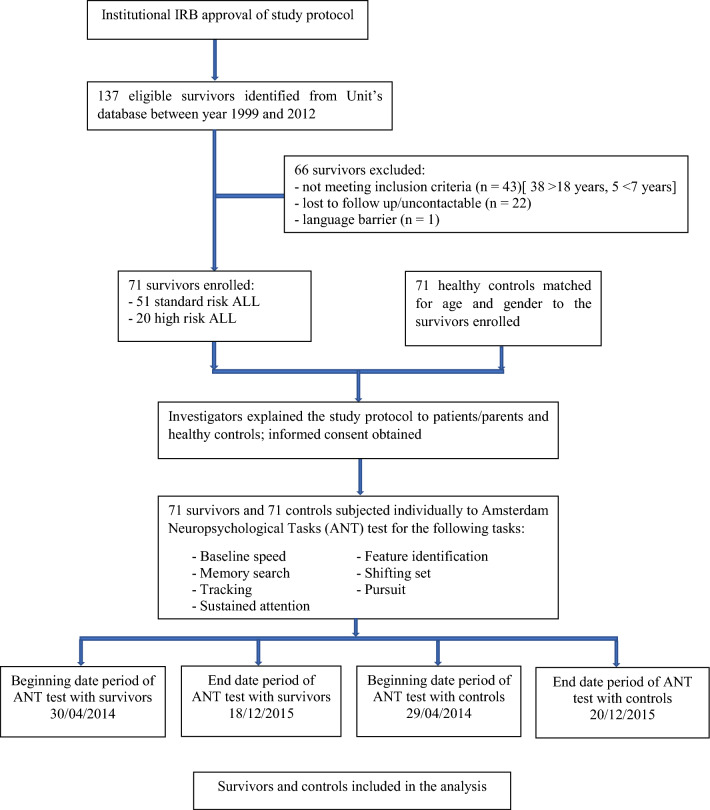
Recruitment of survivors of childhood ALL into the study.

From the database, 137 childhood ALL survivors were identified (Fig. 1). A total of 71 survivors were finally recruited and underwent the ANT assessments with 71 paired healthy controls. Two of the survivors were not able to complete all seven tasks as they became restless and refused to complete the tasks.

Apart from parents’ education level, the demographic data between childhood ALL survivors and healthy controls showed no discernible variations (Table 1). Five of the survivors in the HR group had relapse; four survivors received craniospinal irradiation as part of the treatment protocol (Table 2). High dosage intravenous methotrexate was administered to six patients in the HR group; the mean cumulative dose was 7333.33 mg/m^2^. Meanwhile, a mean cumulative dose of 8100.00 mg/m^2^ and 2000.00 mg/m^2^ of intravenous cytarabine was administered to six patients from the HR group and two patients from the SR group respectively.

**Table 1:** Demographic characteristics of study population.

Characteristic	ALL survivors (n = 71)	Healthy controls (n = 71)	*p* value
Age at study entry in years, mean (SD)	12.18 (3.14)	12.15 (2.99)	0.956
Gender, n (%)			
Male	34 (47.9)	34 (47.9)	1.000
Female	37 (52.1)	37 (52.1)	
Education, n (%)			
Primary school	32 (45.1)	32 (45.1)	0.121
Secondary school	35 (49.3)	39 (54.9)	
Special education	4 (5.6)	0 (0.0)	
Father’s age in years, mean (SD)	47.04 (6.80)	45.49 (6.65)	0.172
Mother’s age in years, mean (SD)	43.63 (6.34)	42.94 (6.20)	0.513
Father’s education level, n (%)			
Primary	7 (9.8)	5 (7.1)	0.005
Secondary	45 (63.4)	28 (39.4)	
Tertiary	19 (26.8)	38 (53.5)	
Mother’s education level, n (%)			
Primary	10 (14.1)	2 (2.8)	0.014
Secondary	38 (53.5)	33 (46.5)	
Tertiary	23 (32.4)	36 (50.7)	
Family’s monthly income, n (%)			
< MYR3000	26 (36.6)	15 (21.1)	0.095
MYR3000-MYR7000	29 (40.9)	32 (45.1)	
> MYR7000	16 (22.5)	24 (33.8)	

*n*, number; SD, standard deviation; MYR, Malaysian Ringgit.

Statistical test: chi-square test for categorical data (Yates correction applied for cell count < 5); student t-test for continuous data; significant value *p* < 0.05.

**Table 2:** Disease and treatment characteristics of survivors of childhood ALL based on risk stratification.

Characteristic	Standard Risk (n = 51)	High Risk (n = 20)	*p* value
Age at study entry in years, mean (SD)	12. 28 (3.17)	13.64 (3.01)	0.104
Age at diagnosis in years, mean (SD)	4.10 (3.17)	5.79 (3.46)	0.003
Male/Female, n (%)	25 (49.0)/26 (51.0)	9 (45.0)/11 (55.0)	0.760
Duration off-treatment in years, mean (SD)	5.47 (3.55)	4.59 (2.95)	0.332
Chemotherapy protocol, n (%)			
UKALL XI	2 (3.9)	0 (0)	0.661
UKALL 97/97(99)	36 (70.6)	13 (65.0)	
UKALL 2003	13 (25.5)	6 (30.0)	
Interfant-99	0 (0)	1 (5.0)	
Cumulative chemotherapy doses in mg/m^2^, mean (SD)			
Dexamethasone	895.58 (501.81)	733.20 (476.85)	0.214
Prednisolone	2506.90 (387.35)	2957.69 (1541.88)	0.320
Cytarabine	1152.94 (235.24)	2352.00 (732.42)	< 0.001
Methotrexate	1825.88 (523.34)	1595.00 (717.59)	0.138
Total injection of intrathecal chemotherapy, mean (SD)*	20.06 (3.68)	23.05 (5.40)	0.009

n, number; SD, standard deviation.

Statistical test: chi-square test for categorical data (Yates correction applied for cell count < 5); student t-test for continuous data; significant value *p* < 0.05.

*Intrathecal methotrexate or cytarabine [monotherapy (dose according to age)] or triple intrathecal therapy with methotrexate, cytarabine and hydrocortisone.

A tabulated result of the tasks below can be found in Supplement (Table 1).

### Baseline speed (BS)

Baseline speed (unit in milliseconds, ms) was derived from a computerized task of alertness. There was no statistically significant difference between the survivors’ and controls’ baseline speed with mean reaction of 339 ± 109 ms and 319 ± 69 ms, respectively (*p* = 0.11). Although the survivors demonstrated more fluctuation in reaction speed with mean SD of 96 ± 87 ms higher than that of the controls’ 77 ± 42 ms, this discrepancy was not statistically significant (*p* = 0.071).

### Memory search letters (MSL)

**Figure 2:**
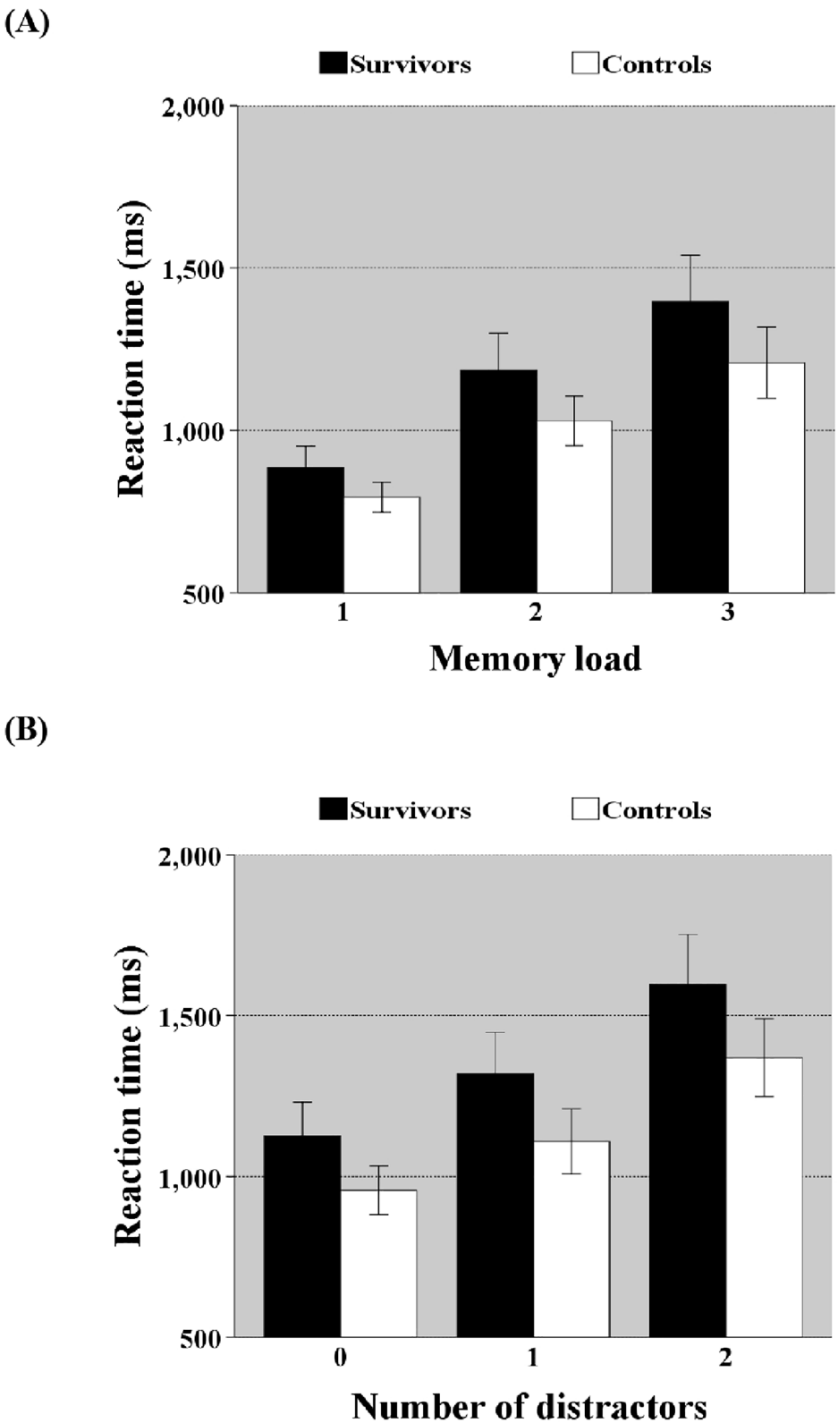
Reaction time ± standard error of mean as a function of memory load (**A**) and distraction (**B**) in task memory search letters (MSL). *Illustrates the significant group × memory load/distraction interaction. The impact of the increase in memory load/distraction is larger in the survivors.*

This letter detection task measured working memory capacity and distraction. In comparison to the controls, the survivors reacted slower [*F*(1,69) = 12.235, *p* < 0.0001, $\eta_{p}^{2}$ = 0.157] and group (controls vs. survivors) interacted with memory load [*F*(2,138) = 3.598, *p* = 0.030, $\eta_{p}^{2}$ = 0.050], implying that the differences in speed between survivors and controls grew as memory load rose (Fig. 2). When the difficulty level of the task was increased (Part 3: less targets, more distractors), data analysis revealed that the groups varied in speed [*F*(1,69) = 16.106, *p* < 0.0001, $\eta_{p}^{2}$ = 0.189] with survivors being slower. Distraction had no effect on the groups (*p* = 0.092) indicating that disparities of speed between survivors and controls did not increase with distraction. Additionally, accuracy varied between groups [*F*(1,69) = 5.747, *p* = 0.019, $\eta_{p}^{2}$ = 0.077], with survivors being less accurate. Group did not interact with distraction (*p* = 0.19), indicating that differences in accuracy did not increase with distraction. The number of errors when the signal contained 0, 1 or 2 distractors) were 1.87 ± 3.85, 3.04 ± 8.22, and 8.66 ± 10.87, respectively for the survivors, and 1.16 ± 2.66, 1.0 ± 2.22, and 5.71 ± 7.20, respectively for the healthy controls. Reaction time (ms) increased as a function of number of distractors (0, 1, 2) from 1123 ± 444, 1314 ± 548, and 1593 ± 664, respectively for the survivors, and 956 ± 324, 1108 ± 434, and 1370 ± 512, respectively for the healthy controls.

### Pursuit (PU) and tracking (TR)

**Figure 3:**
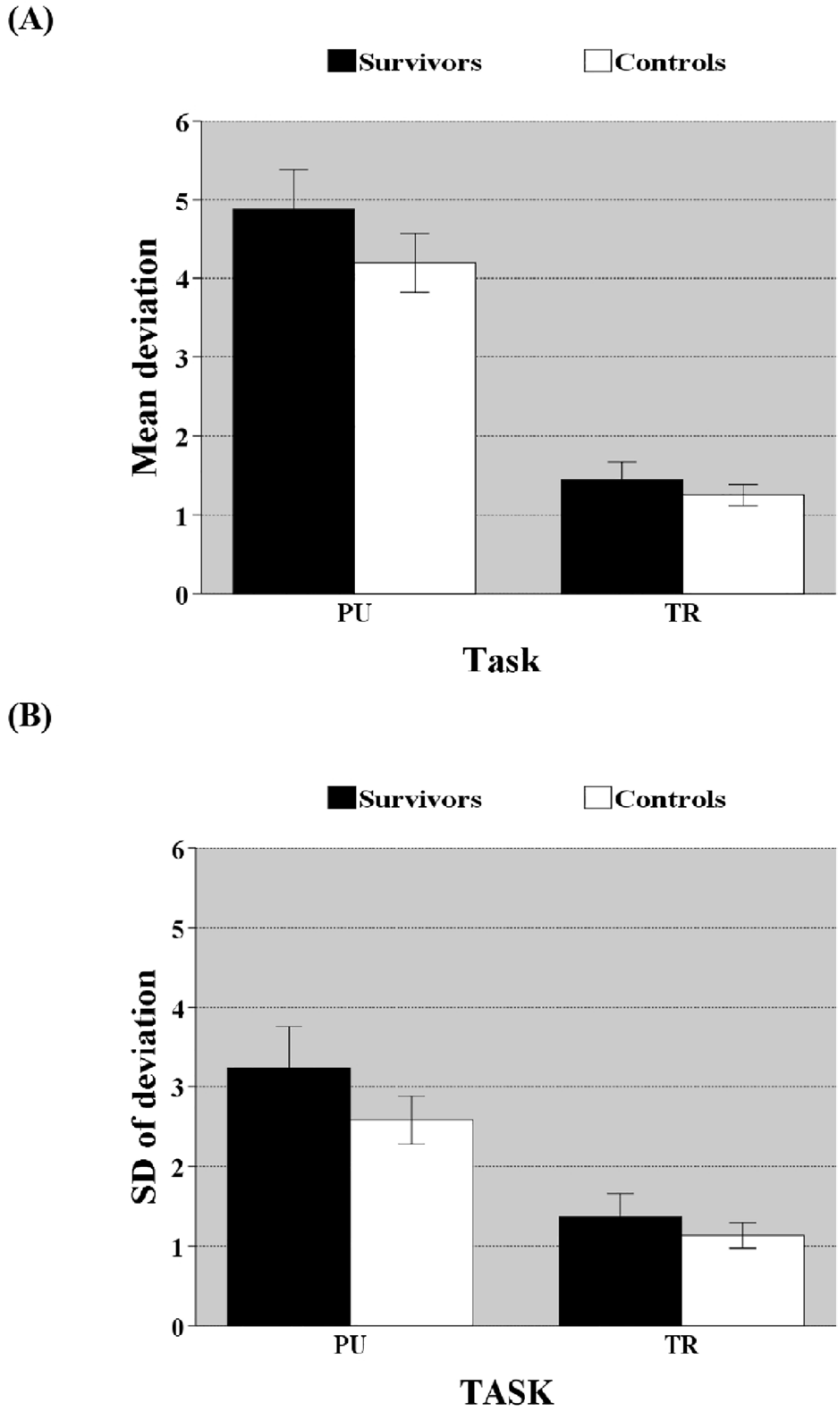
Accuracy ± standard error of mean (**A**) and fluctuation in accuracy ± standard error of mean (**B**) as a function of task [tracking (TR) vs. pursuit(PU)]. *Illustrates the significant group × task interaction. The differences between groups are larger on task PU (higher executive function (EF) demands) than on TR (lower EF demands).*

Tracking measured accuracy and stability of movement along a planned trajectory while pursuit required concurrent planning and execution of movement while tracking a target with random movement. Accuracy varied between groups [*F*(1,68) = 5.817, *p* = 0.019, $\eta_{p}^{2}$ = 0.079] (Fig. 3). Group interacted with task type, with discrepancies between groups larger on PU than on TR [*F*(1,68) = 3.586, *p* = 0.007, $\eta_{p}^{2}$ = 0.102], indicating that when executive function demands were higher, differences between groups increased. Additionally, the survivors also displayed greater fluctuation in accuracy compared to controls [*F*(1,68) = 6.030, *p* = 0.017, $\eta_{p}^{2}$ = 0.081], and group interacted with task type [*F*(1,68) = 5.441, *p* = 0.023, $\eta_{p}^{2}$ = 0.047], with differences on PU being larger than on TR, demonstrating that when executive function demands were higher, distinctions between groups increased.

### Sustained attention dots (SAD)

This task measured the ability to maintain performance at a certain level during a longer period of time. There was a significant difference in tempo and fluctuation in tempo across groups [*F*(1,68) = 9.169, *p* = 0.003, $\eta_{p}^{2}$ = 0.119], [*F*(1,68) = 3.691, *p* = 0.05, $\eta_{p}^{2}$ = 0.052] respectively, but not in error rate (*p* = 0.17). The interaction error type by group was not significant (*p* = 0.39), indicating that error type did not affect outcome. The survivors had a higher fluctuation in tempo (2.35 ± 1.42 s vs. 2.01 ± 1.40 s), and were slower (14.43 ± 6.27 s vs. 12.43 ± 4.97 s) overall.

### Visuo-spatial sequencing (VSS)

**Figure 4:**
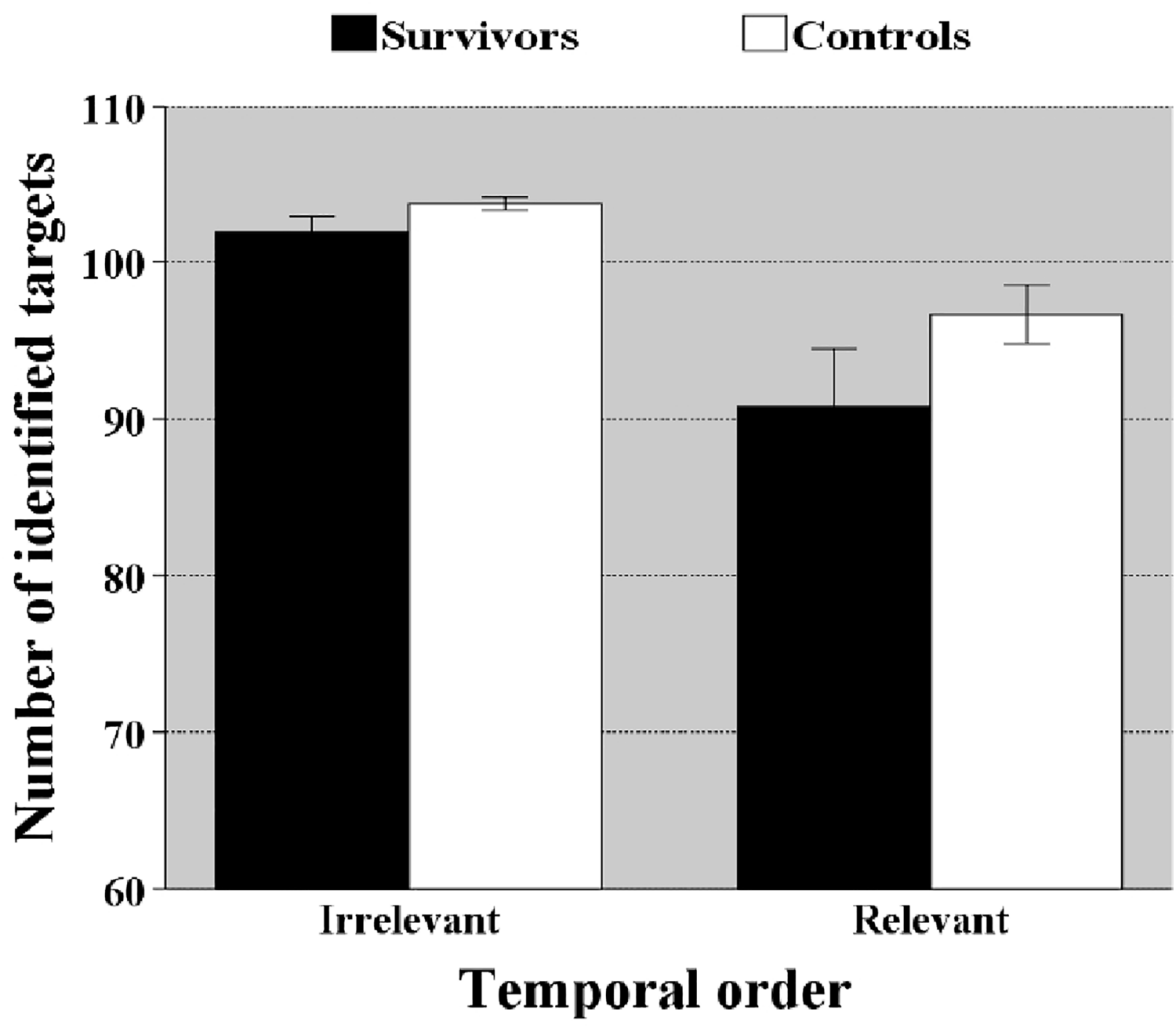
Memory score ± standard error of mean in task visuo-spatial sequencing (VSS) as a function of scoring criterion. *Illustrates the significant group × scoring criterion interaction. When temporal order of the recall is relevant (higher working memory load), the difference between groups is larger.*

This task assessed the subjects’ memory of visuospatial temporal patterns. The number of targets that the control group and survivors were able to identify significantly differed [*F*(1,70) = 8.396, *p* = 0.005, $\eta_{p}^{2}$ = 0.107], and group interacted significantly with Recall criterion [*F*(1,70) = 5.693, *p* = 0.020, $\eta_{p}^{2}$ = 0.075], showing that differences in accuracy is greater when working memory demands were higher (reproduction of visuospatial location *and* temporal order) (Fig. 4).

### Shifting attentional set—visual (SSV)

Two components were assessed in SSV, namely cognitive flexibility and inhibition, which are important for executive functioning.

#### Inhibition

Survivors were generally not slower than controls (*p* = 0.126), and group did not interact with Inhibition (*p* = 0.77). In contrast, more errors were made by survivors in comparison to the controls [*F*(1,69) = 16.043, *p* < 0.0001, $\eta_{p}^{2}$ = 0.189]. However, the interaction was not statistically significant (*p* = 0.124), signifying that differences did not worsen under incompatible conditions.

#### Flexibility

**Figure 5:**
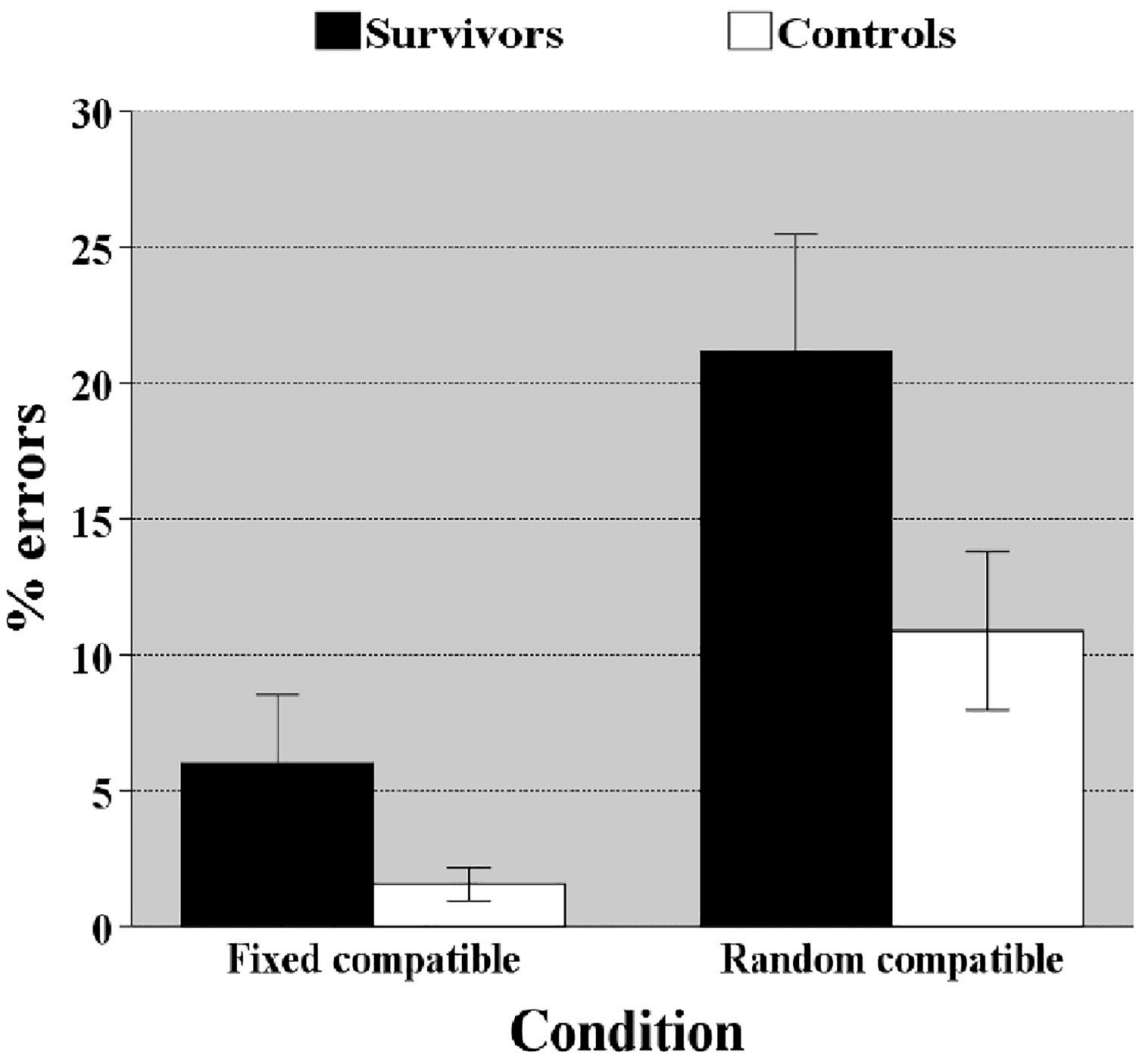
Accuracy ± standard error of mean in task shifting attentional set—visual (SSV) as a function of task condition. *Illustrates the significant group × task condition interaction. Under the random compatible task condition (cognitive flexibility required) the difference between groups is larger than under the fixed compatible condition (flexibility not required).*

There was no difference in speed between groups (*p* = 0.130) and group did not interact with flexibility (*p* = 0.792), suggesting that flexibility demands did not affect outcome differently. Survivors made more errors than controls [F(1,69) = 21.808, *p* < 0.0001, $\eta_{p}^{2}$ = 0.24] and the group × flexibility interaction was significant [F(1,69) = 6.386, *p* = 0.014, $\eta_{p}^{2}$ = 0.085], which indicates that differences in accuracy between groups increased when flexibility was required (Fig. 5).

The overall task performance of the survivors were presented in z-scores when compared to the norm (Table 3), as differences from well-established norms were expressed in standard deviations. This approach is relevant because deviations from the norm are presented according to clinical criteria and the results are independent of the performance level of the group of healthy controls. In ANT, negative z-scores denote better-than-the-norm performance and positive z-scores denote poorer-than-the-norm performance (slower/faster reaction times and higher/lower number of errors result in positive/negative z-scores, respectively). In this study, it can be concluded that overall task performance was practically within the normal range. Visuospatial memory, resistance against distraction, flexibility, and sustained attention were compromised as only between 52 and 63% of the survivors performed within the normal range (z ≤ 1). It was also concluded that the mean performance was in the normal range for 18 out of 20 performance parameters (mean z-score), and the mean performance stayed below the criterion of a severe deficit (z ≥ 2). However, at the individual level, we observed a variable distribution in the severity of deficits. For instance, the percentage of patients exhibiting a severe deficit ranged from 1.4 to 30%. Notably, parameters such as alertness (BS), executive motor control (PU, TR), and memory search (MSL) were found to be relatively spared, with a lower incidence of severe deficits among the study participants. Conversely, sustained attention (SAD), distraction (MSL), inhibition/flexibility (SSV), and visuospatial memory (VSS) were identified as more compromized, with a higher proportion of individuals exhibiting severe deficits in these cognitive domains.

**Table 3:** Percentage of survivors deviating from the norm per task.

Task	Parameters	z ≤ 1	1 < z < 2	z ≥ 2	Mean z
Baseline speed (BS)	Speed	87.0	5.8	7.2	0.12
	Fluctuation	91.3	2.9	5.8	− 0.06
Sustained attention dots (SAD)	Tempo	55.9	26.5	17.6	1.11
	Fluctuation	63.2	23.6	13.2	0.75
	Errors	84.1	5.9	10.0	0.21
Shifting attentional set-visual (SSV)	Overall speed	71.9	14.3	13.8	0.50
	Overall errors	67.6	12.0	20.4	0.95
	Inhibition	74.3	10.0	15.7	0.62
	Flexibility	52.9	17.1	30.0	1.45
Memory search letters (MSL)	Overall speed	85.7	10.0	4.3	-0.07
	Overall errors	81.0	11.2	7.8	0.21
	Load	82.9	14.2	2.9	-0.11
	Distraction	63.3	15.3	21.4	0.55
Pursuit (PU)	Accuracy	76.5	16.1	7.4	0.40
	Fluctuation	91.2	7.3	1.5	0.00
Tracking (TR)	Accuracy	95.7	2.9	1.4	-0.10
	Fluctuation	97.1	1.5	1.4	-0.28
Visuo-spatial sequencing (VSS)	Correct trials	63.0	12.9	24.1	0.64
	Identified targets	77.8	11.1	11.1	0.28
	Targets in correct order	63.0	9.2	27.8	0.79

z ≤ 1 : within normal range or better than the norm.

1 < z < 2 : between 1 and 2 SD below (poorer than) the norm.

z ≥ 2 : more than 2 SD below the norm.

### Relation between task performance and treatment

None of the treatment parameters [age at diagnosis, duration off-treatment, duration on treatment, cumulative IT MTX doses (mg total), cumulative prednisolone/dexamethasone doses and cumulative prednisolone equivalent doses] had significant correlation with any of the ANT performance measures (Table 2 supplement).

## Discussion

The neuropsychological results of the survivors were interpreted in comparison to that of the healthy controls. For baseline motor speed (task BS) and distractability (task MSL), the performance of the survivors of childhood ALL at our center was comparable to that of the healthy controls, which indicate that their alertness level (readiness to respond to an external stimulus) was normal. However, they demonstrated significant neuropsychological deficits in working memory, inhibition and flexibility, sustained attention, executive visuo-motor control, and visuospatial memory compared to the controls. Poorer working memory capacity in the survivors was shown by a larger rise in reaction time after an increase in memory load (task MSL) and a larger fall in memory score after applying a stricter recall criterion (task VSS).

Response inhibition and flexibility (task SSV), both playing an important role in executive functioning, were also significantly affected in our survivors. Without inhibitory control, an individual will be impulsive and unable to finish one task at a time; whereas without flexibility, one faces difficulty juggling multiple tasks and dealing with changing situations or decisions. Deficits in both working memory and executive functioning have been reported among adult ALL survivors’ years after they had been off treatment^18,32^. We did not find any correlation between treatment intensity and the working memory or executive functioning, however a larger cohort study may be required to determine the effect of intensified treatment on the survivors^32,33^.

Impairment in sustained attention among our survivors was demonstrated in the SAD task, which was consistent with studies by Buizer et al.^16^ and Langer et al.^34^. Buizer et al.^16^ reported significant impairment in attention predominantly in those who received intensified treatment whereas Langer et al.^34^ reported significantly poorer performance in survivors who received CI. Buizer et al.^15^ and Knight et al.^23^ both reported significantly worse visuo-motor control (tasks pursuit and tracking) among the ALL survivors compared to healthy controls, signifying deficit in higher cognitive function. Schuitema et al.^35^ also reported similar observation, 25 years after completion of treatment, with patients who received CI during treatment performing poorer compared to those on chemotherapy alone. Female sex and longer duration from treatment completion were associated with worse performance, however age at diagnosis was not found to influence the outcome^15^. Our survivors also experienced deficit in visuo-motor control, however we did not identify any factors associated with the deficit.

The frontal and prefrontal cortices of the brain are involved in mediating working memory and executive functioning, thus play an important part in learning processes and carrying out activities of daily living^36–38^. Sustained attention was controlled by the prefrontal and parietal cortices^39^ whereas visuo-motor function was linked with the frontal, parietal, and temporal white matter tracts^35^. As the myelination of frontal, prefrontal cortex and cerebellar-prefrontal networks takes a protracted course during childhood, the less mature brain in younger children is more susceptible to damage by CNS-directed therapy^33,40,41^.

The effect of duration off-treatment on the neuropsychological outcome remains controversial. While patients receiving CI showed a definite decline in performance with time, studies on neuropsychological outcomes for those receiving chemotherapy alone were less robust. We postulate that after an acute insult to the brain following chemotherapy, there may be a period of gradual catch-up due to neural plasticity of an immature brain leading to these improvements. However, it remains to be seen whether this improvement can be sustained over time and with the increasing environmental demands. Due to the short off-treatment duration in our cohort, a follow-up study would be informative to examine the long-term outcome in our survivors.

Bisen-Hersh et al.^42^ highlighted academic difficulties among the survivors of childhood ALL, emphasizing on impairment in attention, working memory and processing speed. They concluded that these impairments were significant with neurophysiological and pre-clinical evidence indicating white matter abnormalities and acquired brain damage from intravenous and IT methotrexate and CI^42^. Krull et al.^43^ reported that 2 decades after diagnosis, neurocognitive impairment was prevalent among the survivors receiving lower dose CI, as well as those who received chemotherapy only. This neurocognitive impairment affects their functional outcomes when they return to school after cancer treatment, and later in adult life when they graduate and seek full-time employment^43,44^. Survivors of childhood leukemia were reported to have lower grades in school, especially those with younger age at diagnosis^44^. Unfortunately, our study may not be powered to detect the impact of younger age at diagnosis on task performance. With the current education approach in Asian countries which revolves around memorization and exam-orientated learning, it is important for us to recognize that these survivors will experience hardship to catch-up with the peers due to their neuropsychological deficits.

This study had several limitations. An attempt to match the healthy controls to the survivors based on socio-economic status was not feasible as the controls were recruited from schools around the suburban/city area. Our study included a mixture of participants who underwent chemotherapy-only and combined therapy (CI). While this approach allowed for a broader representation of survivors, it complicates the interpretation of treatment effects as they introduce heterogeneity, making it challenging to attribute observed cognitive outcomes solely to one form of treatment. Correlation analysis between task performance and certain treatment parameters for example CI and cumulative chemotherapy (high dose methotrexate/cytarabine) doses was not performed due to the small number of survivors in these group. Therefore, we were unable to determine any significant association between these variables and neuropsychological deficits. Additionally, in interpreting the results of our study, the norms utilized for the ANT were derived from a Dutch population. While the ANT is a widely employed cognitive assessment tool, the cultural and demographic variations between the reference population and our study participants may cause a degree of variability in the interpretation of individual performance. The wide age range of 7–18 may have caused potential variability in cognitive outcomes due to developmental differences. Furthermore, the small sample size may restrict the statistical power needed to detect subtle effects. While we aimed to capture a more diverse range of survivors, the limitations between inclusivity and statistical power should be acknowledged.

We conclude that survivors of childhood ALL in our center showed significant neuropsychological deficit compared to healthy controls. The major domains of deficit were in working memory, sustained attention, and executive functioning (in particular attentional flexibility). It is essential to develop treatment protocols that are effective but less harmful, such as targeted delivery drugs, to preserve neuropsychological function in survivors of childhood ALL.

## Methods

This single-center, case-control study was conducted in Pediatric Hematology and Oncology Unit at Department of Pediatrics, Faculty of Medicine, The National University of Malaysia (UKM) over a 2-year period. Ethical approval was acquired from the Research Ethics Committee Universiti Kebangsaan Malaysia and the study was conducted in accordance with the Declaration of Helsinki. Written informed consent was taken from the parents prior to participation.

### Treatment protocols

The chemotherapy protocols used for the treatment of childhood ALL in UKM were modified UKALL XI in 1999–2000, modified UKALL97 or 97(99) during 2001–2008, and modified UKALL2003 from year 2009 onwards. Patients on modified UKALL 97, 97(99) or modified 2003 protocol were assigned to either standard risk (SR) (Regimen A) or high risk (HR) (Regimen B and C), based on their risk stratification. Prednisolone and dexamethasone were used in the modified UKALL97(99) and UKALL2003 respectively, whereas 6-mercaptopurine was used in both modified protocols. All other chemotherapy drugs and treatment duration followed the original Regimen A, B, and C protocols. Chemotherapy drugs consisted of: intravenous vincristine, daunorubicin, cyclophosphamide, doxorubicin; intramuscular *E. coli* asparaginase; subcutaneous cytarabine; oral prednisolone or dexamethasone, oral methotrexate, oral 6-mercaptopurine and intrathecal methotrexate ± intrathecal cytarabine. All patients with relapse in this study received modified ALL-REZ-BFM 90 protocol. Craniospinal irradiation (24 Gy in 15 fractions of 1.6 Gy each) was given to patients with CNS relapse. Meanwhile, patients diagnosed with infantile leukemia received modified Interfant-99 protocol.

### Subjects

Childhood ALL survivors aged 7–18 years, who had completed their treatment for a minimum of 1 year and remained in remission were eligible for the study. This cohort of survivors could inevitably include some children whose school performances were below average before their illness and possibly remained below average even without the impact of ALL and its treatment. Evidence have shown weak to no correlation between ANT task performance and intelligence (i.e., IQ measure) in children^33–36^.

A control group of healthy school children was randomly selected from schools in Kuala Lumpur based on the following criteria: no known medical illness, average to good school performance as per school reports, good school attendance, same schooling year of patients and same gender. As the performance on the selected neuropsychological tasks, i.e., reaction time tasks was known to be affected by age and gender^32,33^, the healthy controls were matched to the survivors based on these two parameters. A ‘match' was defined as individuals within the same calendar year of birth and of the same biological gender.

In ANT tasks, the requested answer was a keypress on the basis of a simple instruction. The tasks were language-culture-free and the possibility that education or social circumstances affecting the results was minimal.

In selecting the age range for our study, we chose to include subjects aged 7 and above due to the educational context in Malaysia. At the age of 7, students typically commence their primary education, specifically in Primary 1. At this stage, students are expected to possess foundational skills such as basic reading abilities and numerical knowledge. By focusing on this age range, we aimed to capture a cohort that has undergone initial educational exposure, ensuring a baseline level of cognitive development and readiness for the tasks involved in our study. Consequently, subjects below the age of 7 were excluded to maintain consistency with the educational milestones and developmental expectations associated with the early years of formal education in Malaysia. We also restricted the upper age limit to 18 years to ensure that only individuals within the pediatric age group were covered, considering the distinctive challenges and considerations associated with pediatric cancer survivorship. This age range is also consistent with the typical age group used in most pediatric oncology studies to facilitate comparability across research findings.

### Tool

The Amsterdam Neuropsychological Tasks (ANT) program is a computerized neuropsychological test used to assess fundamental processes that underlie the execution of advanced cognitive processes, i.e., alertness, sustained attention, working memory, inhibition, cognitive flexibility, and visuomotor control^32^. The ANT program uses task paradigms in which task demands are manipulated. For example, increasing memory load in a memory search task causes an increase in reaction time and/or number of errors, i.e., the task effect. By comparing the task effects between a control and the study group, the manipulated process (working memory) can be used to interpret the disparities between these groups. It enables precise assessment of the speed and accuracy of certain components of executive function and attention^32^. This program has been used in different clinical diagnoses, known to cause a diffuse impact on brain function, with satisfactory sensitivity and validity, such as ADHD, multiple sclerosis, phenylketonuria, and neurofibromatosis^37–42^. Buizer et al.^13^ and Schuitema et al.^14^ used ANT to examine neuropsychological outcomes of childhood leukemia following treatment with chemotherapy, or craniospinal irradiation and chemotherapy respectively^22,43^. We have also used the ANT program to evaluate neuropsychological outcome among survivors of childhood brain tumor in our center^44^.

In the current study, we selected seven tasks, administered in a fixed order: baseline speed (BS), memory search letters (MSL), sustained attention dots (SAD), tracking (TR), pursuit (PU), shifting attentional set–visual (SSV), and visuo-spatial sequencing (VSS). These tasks allowed us determine a more specified profile of neuropsychological (dys)functioning, including alertness (BS), working memory (MSL, VSS), sustained attention (SAD), inhibition and cognitive flexibility (SSV), and executive visuomotor control (PU, TR). The ANT tasks’ validity and test–retest reliability were both good and well-documented^42^. Detailed tasks descriptions can be found in the supplement.

The investigators completed in-depth training on how to conduct the ANT tasks prior to the study commencement. All tests were conducted consistently in the morning in a quiet room at the clinic. A laptop screen was used to display all test stimuli. Participants had to respond by clicking a mouse button or using the mouse as a tracking device. For right-handed participants, the right and left mouse buttons were designated as ‘Yes’ and ‘No’ responses respectively, and vice versa for left-handed participants. Each test was preceded by a briefing, during which the participants received verbal instructions as well as a visual presentation of the different probe and stimulus types. This was followed by two practice chances for each activity to ensure that they understood the instructions well. In general, participants were told to respond as quickly and precisely as they could while performing the tests. The outcome variables for all reaction time (RT) tasks were speed and accuracy of responses for each signal type; unless otherwise stated, the employed post-response interval (PRI: duration between responses and next stimulus start) was 1200 ms and signals were given in pseudo-random sequence^38^.

### Data analysis

Tests of homogeneity of variance and normality were conducted and assumptions were identified. GLM repeated measures ANOVA(RM-ANOVAs) were used to analyze the results. As each survivor was individually paired with a control subject, Group (controls vs. survivors) was entered as within-subject (WS) factor, using the SPSS program version 27. RM-ANOVAs were run by inputting the mean speed and fluctuation in speed in task BS, mean tempo, fluctuation in tempo, mean error rate, and post-error slowing in task SAD, respectively. As values of a second WS factor, different task manipulations (see supplement) were recorded for each task. MSL: memory load (parts 1–3), distraction within part 3 (0–2 distracters); SAD: bias (misses vs. false alarms); SSV: attentional flexibility [not-required (part 1)], —required (part 3, compatible trials), and inhibition [compatible (part 1)], incompatible (part 2); VSS: recall criterion (correct order relevant vs. irrelevant). Task type (TR vs. PU) was entered as a WS factor, while mean deviation and fluctuation in deviation were inputted in RM-ANOVAs. The group × task manipulation interactions was the main focus in the RM-ANOVAs.

Correlational evaluations were conducted to investigate the potential relationships between task performance (using z-scores generated by the program) and age at diagnosis, gender, on and off treatment duration, and treatment intensity. The regression functions are based on norm samples of participants with normal development, including 6.770 (BS), 3.240 (MSL), 2.340 (PU), 3.260 (TR), 3.180 (SAD), 830 (VSS), and 3.440 (SSV). As a result, they are regarded as reliable predictions of performance level^33^. The survivors were divided into of the following three categories: z ≤ 1 (within the normal range or better than the norm), 1 < z < 2 (between 1 and 2 SD deviating from the norm: mild impairment), z ≥ 2 (more than 2 SD deviating from the norm: severe impairment)^43^.

Chi-square test was used to analyze categorical data (Table 1), which were reported in frequency and percentage. Data that were not normally distributed were analyzed using Mann–Whitney U test. A significant *p*-value was determined at < 0.05. Effect sizes were computed using partial eta squared with $\eta_{p}^{2}$ ∼ 0.03 denoting a weak effect, $\eta_{p}^{2}$ ∼ 0.06 denoting a moderate effect and $\eta_{p}^{2}$ ≥ 0.14 considerably a large effect^50^.

### Supplementary Information

Supplementary Information.

## Data Availability

The datasets used and/or analyzed during the current study available from the corresponding author on reasonable request.
