# Supplementary material for: Neuropsychological task outcomes among survivors of childhood acute lymphoblastic leukemia in Malaysia
Source: Sci Rep. 2024 Apr 4;14:7915. doi: 10.1038/s41598-024-58128-1 (PMC10995164; doi:10.1038/s41598-024-58128-1)
Supplement: Supplementary file 1 — Supplementary Information. [file 41598_2024_58128_MOESM1_ESM.docx]

Amsterdam Neuropsychological Task (ANT)

Baseline speed (BS)

This task measures alertness which requires minimal cognitive effort, as other information about the stimulus, except its arrival (presence) is not necessary^1^. Subjects were required to press a mouse-key with the index finger as quickly as possible when a fixation cross in the centre of the computer screen changed into a white square. Post response interval until the next stimulus presentation varied randomly between 500 and 2500 ms to prevent anticipation strategies. The task consisted of two parts with 32 trials for the preferred hand and non-preferred hand. The mean speed and within-subject SD of reaction times (fluctuation in speed) were calculated over both the preferred and the non-preferred hand responses.

Memory search letters (MSL)

This letter detection task measures working memory capacity and distraction^2^. The display set of four letters consists of three parts with increasing memory load from one item in part 1 (k), to two items in part 2 (k+r), and three items (k+r+s) in part 3. Subjects were required to press the ‘yes’-button to a *complete* target set and the ‘no’-button to incomplete target sets. Target letters in non-target trials act as distractors. Reaction time to target signals is predicted to increase linearly with memory load, reflecting the prolongation of the memory search stage, with the slope of reaction time denoting the rate of memory search. In part 3, the presence of an insufficient number of target letters in non-target signal affects response time in that reaction time and number of errors will increase with the number of ‘distracters’ (0, 1, or 2). Each part consists of 50% target and 50% non-target signals with 40, 72, and 96 trials in parts 1–3, respectively, with non-target trials evenly divided across distracter type (20 ‘0 distractor’ trials in part 1, 18 ‘0’ and ‘1’trials in part 2, and 16 ‘0’, ‘1’ and ‘2’ trials in part 3. Whether an increase in memory load and distraction differentiates between controls and survivors will require a statistically significant interaction between group and task manipulation.

Sustained attention dots (SAD)

This task measures the ability to maintain performance at a certain level during a longer period of time. During this task 600 random patterns of three, four or five dots are successively presented in 50 series of 12 trials. Subjects are required to respond to the 4-dots pattern (target) by pressing the mouse button with their preferred hand (‘yes’-response) and to the 3- or 5-dots patterns (non-targets) by pressing the mouse with their non-preferred hand (‘no’-response). The ratio targets/non-targets is 1/2 which invokes a response bias for the ‘no-response.’ Failure to inhibit this bias is expected to result in the production of relatively more misses than false alarms. Main outcome measures are the 50 series completion times, and the number and type of errors per series. Tempo is operationalised as the mean of the 50 completion times (in seconds). Fluctuation in tempo is operationalised as the within-subject standard deviation of the 50 completion times (in seconds). The primary measure of sustained attention is the within-subject SD of the completion times of the 50 series. Whether failure to sustain inhibition of this bias differentiates between controls and survivors will require a statistically significant interaction between group and error type (misses vs. false alarms). During performance, subjects were informed about errors by a beep signal. The task model predicts that feedback results in post-error slowing. Correct responses following an error were separately registered enabling the measurement of the effect of feedback. Post-error slowing is defined as the difference RT_afterfeedback_ – RT_regular_, with minor slowing suggesting lack of behavioural control (impulsivity).

Tracking (TR)

This task measures accuracy and stability of movement along a planned trajectory. The subject is required to trace the mouse cursor in between the inner circle (radius 7.5 cm) and outer circle (radius 8.5 cm) presented on the computer screen. The cursor had to be moved in clockwise direction once with the right hand and once in counter-clockwise direction with the left hand. The trajectory was divided into 60 radially equal segments and the program computed the mean distance between the cursor trajectory and the midline per segment, resulting in 60 deviation scores. Mean (absolute) deviation of the moving target (accuracy of movement) from the midline and the within-subject SD of the 60 deviation scores (stability of movement) during TOT were taken as main outcome parameters.

Pursuit (PU)

This task demands the concurrent planning and execution of movement. It assesses the quality of executive motor control. Subject is required to continuously track a target moving randomly on the screen, by moving the computer mouse as closely as possible. The task time is 60 s. The program calculates the mean distance between the mouse and the target per second task time, resulting in 60 deviation scores. Main outcome parameters are the mean distance to the target and the within-subject SD of the 60 deviation scores. Task PU places higher executive control demands compared to task TR as movement direction cannot be planned ahead. The results of PU and TR will be analysed together. Whether an increase executive demand differentiates between controls and survivors will require a statistically significant interaction between group and task type (TR vs. PU).

Shifting attentional set – visual (SSV)

This task assesses cognitive flexibility and inhibition. A coloured square moves randomly to the right and to the left of a horizontal bar that is permanently present on the computer screen. The task consists of three parts. In part 1 (fixed compatible condition) the subject is asked to follow the movement of a green square by pressing the left button upon a left move and the right button upon a right move. In part 2 (fixed incompatible condition), using a red square, the subject is asked to do the opposite, by pressing the left button upon a right move and vice versa, requiring the inhibition of prepotent responses. Inhibition is operationalized as the contrast in performance (speed/accuracy) between part 1 and part 2. In part 3 (random condition), the block changes colour randomly requiring the child to follow or ‘mirror’ the movement, depending on the colour of the block. Now the subject needs to shift response sets, i.e., readily switch between execution of a prepotent response and inhibition of a prepotent response, a switch requiring cognitive flexibility. Cognitive flexibility is operationalized as the contrast in performance between part 1 and part 3 (compatible trials). It is expected that the cost of inhibition and flexibility results in slower responses and/or more errors. Whether inhibition and/or flexibility demands differentiate between controls and survivors will require statistically significant interactions between group and inhibition or flexibility, respectively.

Visuo-spatial sequencing (VSS)

This task evaluates memory of visuospatial temporal patterns. In each trial, several circles are pointed out in an array of nine circles, arranged in a 3 × 3 matrix on the computer screen. The subject has to point out the same circles in the same order by moving the mouse cursor to the right locations and perform a mouse-click on each of them. After the subject says that he is ready, the tester starts a new trial. The test consists of 24 trials in which the number of target circles varies from three to seven and in which the spatial sequential patterns increase gradually in complexity. Main outcome variables are the number of correctly completed trials, the total number of correctly identified circles, and the total number of correctly identified circles in the correct order. The task model predicts that the latter number is smaller than the former, as not only the spatial locations but also the temporal sequence must be reproduced (higher working memory demands. Whether inhibition and/or flexibility demands differentiate between controls and survivors will require a statistically significant interaction between group and (scoring) criterion (temporal sequence irrelevant = lenient criterion vs. relevant = strict criterion).

Examples of stimuli and timing between signals can be found in Koekkoek et al.^3^ for tasks BS, PU and TR, for tasks SAD, MSL, and SSV in De Sonneville et al.^2^, and for task VSS in Schuitema et al.^4^. Test–retest reliability and validity of the ANT are satisfactory and have extensively been described^2,5-7^. Mean test–retest reliability for the variables varies, depending on task between 0.73 and 0.80^5^.

Table 1: Task performance between survivors and healthy controls

| **Task** | **Condition** | **Mean (SD)** | | **F** | ***p*** | **$\eta_{p}^{2}$** |
| --- | --- | --- | --- | --- | --- | --- |
|  |  | **ALL** | **Control** |  |  |  |
| BS | Speed | 339 (109) | 319 (69) | 2.427 | 0.11 | 0.017 |
|  | Fluctuation | 96 (87) | 77 (42) | 3.367 | 0.071 | 0.047 |
| SAD | Tempo | 14.43 (6.27) | 12.43 (4.97) | 9.169 | 0.003 | 0.119 |
|  | Fluctuation | 2.35 (1.42) | 2.01 (1.40) | 3.691 | 0.005 | 0.052 |
| PU | Accuracy | 4.87 (2.12) | 4.23 (1.58) | 5.817 | 0.019 | 0.079 |
|  | Fluctuation | 3.23 (2.22) | 2.54 (1.15) | 6.030 | 0.017 | 0.081 |
| TR | Accuracy | 1.44 (0.92) | 1.26 (0.57) | 7.691 | 0.007 | 0.102 |
|  | Fluctuation | 1.36 (1.20) | 1.15 (0.67) | 5.441 | 0.023 | 0.074 |
| VSS | Numbers correct | 101.9  (4.69) | 103.79 (1.76) | 8.396 | 0.005 | 0.107 |
|  | Targets in correct order | 90.77 (15.57) | 96.49  (7.86) | 5.693 | 0.020 | 0.075 |
| SSV | Inhibition | 6.00  (10.56) | 1.54  (2.56) | 16.043 | <0.0001 | 0.189 |
|  | Flexibility | 21.18 (18.02) | 10.50 (12.00) | 6.386 | 0.014 | 0.085 |
| MSL | Speed (Part 1) | 884.48 (282.60) | 793.41 (196.38) | 12.235 | <0.0001 | 0.157 |
|  | Speed (Part 3) | 1123.80 (444.74) | 956.20 (323.52) | 16.106 | <0.0001 | 0.189 |
|  | Accuracy | 1.87 (3.58) | 1.16 (2.67) | 5.747 | 0.019 | 0.077 |

Table 2: Correlation between task performance and treatment parameters

| **Tasks** | **Parameters** | **Pearson correlation** | ***p* value** |
| --- | --- | --- | --- |
| BS  Speed | Age at diagnosis  Duration on treatment  Duration off treatment  Total IT MTX  Cumulative prednisolone equivalent dose | 0.062  -0.091  -0.078  -0.051  0.012 | 0.615  0.457  0.522  0.679  0.925 |
| BS  Fluctuation | Age at diagnosis  Duration on treatment  Duration off treatment  Total IT MTX  Cumulative prednisolone equivalent dose | 0.003  -0.164  0.007  -0.145  -0.120 | 0.983  0.179  0.956  0.234  0.327 |
| SAD  Tempo | Age at diagnosis  Duration on treatment  Duration off treatment  Total IT MTX  Cumulative prednisolone equivalent dose | -0.042  0.058  -0.075  -0.061  -0.083 | 0.736  0.640  0.548  0.627  0.503 |
| SAD  Fluctuation | Age at diagnosis  Duration on treatment  Duration off treatment  Total IT MTX  Cumulative prednisolone equivalent dose | -0.174  -0.124  0.002  -0.161  -0.116 | 0.158  0.319  0.988  0.194  0.351 |
| SSV  Inhibition | Age at diagnosis  Duration on treatment  Duration off treatment  Total IT MTX  Cumulative prednisolone equivalent dose | 0.044  -0.175  0.161  -0.222  -0.144 | 0.720  0.147  0.183  0.065  0.236 |
| SSV  Flexibility | Age at diagnosis  Duration on treatment  Duration off treatment  Total IT MTX  Cumulative prednisolone equivalent dose | 0.002  -0.178  0.268  -0.277  -0.165 | 0.988  0.141  0.025*  0.020*  0.172 |
| PU  Accuracy | Age at diagnosis  Duration on treatment  Duration off treatment  Total IT MTX  Cumulative prednisolone equivalent dose | -0.109  0.123  -0.133  0.059  0.119 | 0.376  0.317  0.280  0.631  0.334 |
| PU  Stability | Age at diagnosis  Duration on treatment  Duration off treatment  Total IT MTX  Cumulative prednisolone equivalent dose | -0.059  0.072  -0.134  0.109  0.142 | 0.635  0.559  0.276  0.375  0.249 |
| TR  Accuracy | Age at diagnosis  Duration on treatment  Duration off treatment  Total IT MTX  Cumulative prednisolone equivalent dose | -0.172  0.135  -0.036  -0.027  0.024 | 0.157  0.268  0.766  0.823  0.846 |
| TR  Stability | Age at diagnosis  Duration on treatment  Duration off treatment  Total IT MTX  Cumulative prednisolone equivalent dose | -0.122  -0.027  -0.149  0.026  0.067 | 0.317  0.827  0.221  0.833  0.586 |
| VSS  Identified targets | Age at diagnosis  Duration on treatment  Duration off treatment  Total IT MTX  Cumulative prednisolone equivalent dose | -0.157  -0.026  -0.175  -0.126  0.124 | 0.256  0.853  0.206  0.362  0.371 |
| VSS  Targets in correct order | Age at diagnosis  Duration on treatment  Duration off treatment  Total IT MTX  Cumulative prednisolone equivalent dose | -0.077  -0.128  -0.173  -0.212  0.011 | 0.579  0.356  0.212  0.123  0.937 |

*correlation is significant at the 0.01 level (2-tailed)

**References**

1 Konrad, K., Gunther, T., Hanisch, C. & Herpertz-Dahlmann, B. Differential effects of methylphenidate on attentional functions in children with attention-deficit/hyperactivity disorder. *J Am Acad Child Adolesc Psychiatry* **43**, 191-198, doi:10.1097/00004583-200402000-00015 (2004).

2 De Sonneville, L. M. *et al.* Information processing characteristics in subtypes of multiple sclerosis. *Neuropsychologia* **40**, 1751-1765, doi:10.1016/s0028-3932(02)00041-6 (2002).

3 Koekkoek, S. *et al.* Effects of highly active antiretroviral therapy (HAART) on psychomotor performance in children with HIV disease. *Journal of neurology* **253**, 1615-1624, doi:10.1007/s00415-006-0277-x (2007).

4 Schuitema, I. *et al.* Executive Dysfunction 25 Years after Treatment with Cranial Radiotherapy for Pediatric Lymphoid Malignancies. *J Int Neuropsychol Soc* **21**, 657-669, doi:10.1017/s1355617715000788 (2015).

5 de Sonneville, L. *Handboek ANT [Handbook ANT]*. 347-399 (Boom Publishers, 2014).

6 Günther, T., Herpertz-Dahlmann, B. & Konrad, K. [Reliability of attention and verbal memory tests with normal children and adolescents--clinical implications]. *Z Kinder Jugendpsychiatr Psychother* **33**, 169-179, doi:10.1024/1422-4917.33.3.169 (2005).

7 Rowbotham, I., Pit-ten Cate, I. M., Sonuga-Barke, E. J. & Huijbregts, S. C. Cognitive control in adolescents with neurofibromatosis type 1. *Neuropsychology* **23**, 50-60, doi:10.1037/a0013927 (2009).
